# Genome Walking by Next Generation Sequencing Approaches

**DOI:** 10.3390/biology1030495

**Published:** 2012-10-01

**Authors:** Mariateresa Volpicella, Claudia Leoni, Alessandra Costanza, Immacolata Fanizza, Antonio Placido, Luigi R. Ceci

**Affiliations:** 1 Department of Biosciences, Biotechnology and Pharmacological Sciences, University of Bari, Via Amendola 165/A, 70126 Bari, Italy; Email: mariateresa.volpicella@biologia.uniba.it (M.V.); cla.leoni@biologia.uniba.it (C.L.); alessandra.costanza@biologia.uniba.it (A.C.); fanimma@libero.it (I.F.); antonio.placido@biologia.uniba.it (A.P.); 2 Institute of Biomembranes and Bioenergetics, Italian National Research Council (CNR), Via Amendola 165/A, 70126 Bari, Italy

**Keywords:** genome walking, insertional mutagenesis, next generation sequencing, functional genomics, gene therapy, transposon tagging, 454-pyrosequencing, Illumina, SOLiD, Ion Torrent

## Abstract

Genome Walking (GW) comprises a number of PCR-based methods for the identification of nucleotide sequences flanking known regions. The different methods have been used for several purposes: from *de novo* sequencing, useful for the identification of unknown regions, to the characterization of insertion sites for viruses and transposons. In the latter cases Genome Walking methods have been recently boosted by coupling to Next Generation Sequencing technologies. This review will focus on the development of several protocols for the application of Next Generation Sequencing (NGS) technologies to GW, which have been developed in the course of analysis of insertional libraries. These analyses find broad application in protocols for functional genomics and gene therapy. Thanks to the application of NGS technologies, the original vision of GW as a procedure for walking along an unknown genome is now changing into the possibility of observing the parallel marching of hundreds of thousands of primers across the borders of inserted DNA molecules in host genomes.

## 1. Introduction

The identification of unknown nucleotide sequences starting from a previously identified DNA region can be directly obtained by a number of Genome Walking (GW) methods all having in common a final PCR amplification in which an oligonucleotide specific for the known sequence is coupled with an oligonucleotide derived from the adopted GW strategy. The numerous GW methods can be classified into three main categories, according to the first step of the whole strategy: (1) **Restriction-based GW methods**, requiring a restriction digestion of genomic DNA and ligation of restriction fragments to DNA-cassettes; (2) **Primer-based GW methods**, in which PCR amplifications are directly carried out using a variously designed combinatorial (random or degenerate primer) coupled to a sequence specific primer; (3) **Extension-based GW methods**, in which the extension of a sequence specific primer and subsequent 3'-tailing of the resulting single strand DNA (ssDNA) provide the substrate for the final PCR amplification. Critical overviews of the conceived GW strategies, and their possible applications to both eukaryotic and prokaryotic genomes, have been recently reported [1,2,3].

GW is a highly flexible approach, allowing both the identification of specific, unique sequences (as for the analysis of single gene flanking sequences) and the analysis of large libraries (such as those obtained by insertional mutagenesis of retroviruses and transposable elements). In the latter case, GW has the potential to be powered by the enormous capacity of Next Generation Sequencing (NGS) approaches. In this review we focus on the application of NGS to GW. Among NGS technologies [4,5], currently only pyrosequencing and the sequencing by synthesis (SBS) methods have been reported as successful sequencing approaches for GW. A first report on the application of pyrosequencing (using the Roche 454 platform) to GW is that of Wang *et al.* [6], while the first application of SBS methods (using the Illumina technology) to GW is more recent [7]. Nowadays both technologies have been applied to GW by several research groups and, recently, specific methodological papers have been published [8,9,10]. In principle, however, also other NGS technologies in which parallel sequencing of amplicons is performed (such as the SOLiD “Sequencing by Oligo Ligation Detection” approach and the more recent “sequencing by synthesis” approach, known as Ion Torrent [11], by Life Technologies), can potentially be used for GW.

The first part of this review illustrates applications of pyrosequencing to GW, describing protocols adopted for the analysis of genomes of man, mouse, yeasts and plants, then the application of the SBS-Illumina technology to GW is reported. A final paragraph gives some general considerations about the possibility to use other, as yet unexplored, combinations of NGS and GW methods.

## 2. GW by 454-Pyrosequencing

**Figure 1:**
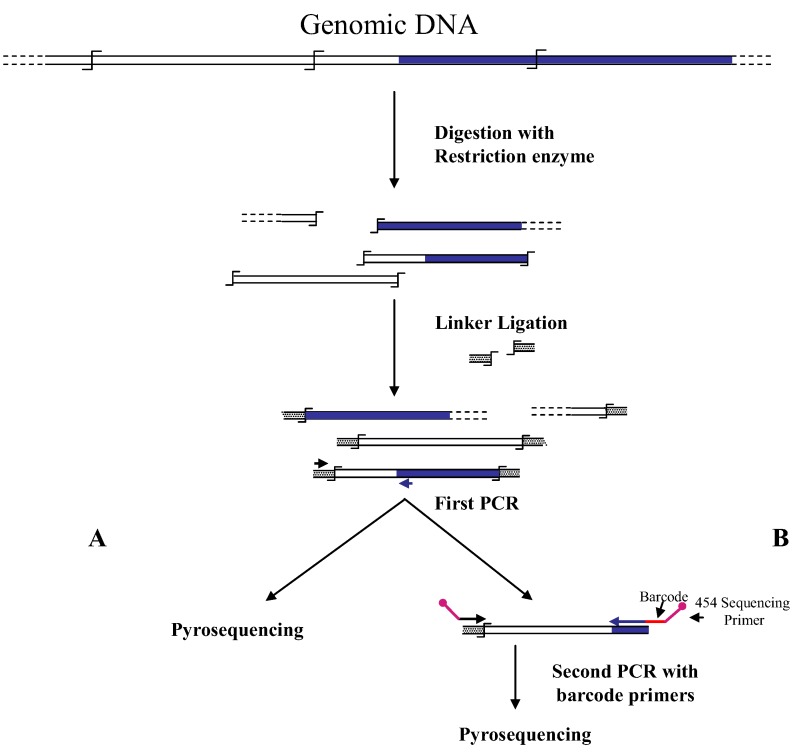
Main steps of cassette-PCR genome walking (GW) and pyrosequencing association strategies. The original strategy reported by Wang *et al.* [6] (**A**) and its improvement by the adoption of barcoded primers [13] (**B**) are illustrated. Blue and white bars correspond to known and unknown sequences, respectively. A black horizontal arrow indicates adapter specific primers; a blue arrow represents primers designed in the known region. The 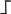 symbol indicates a restriction site. Barcodes and 454 sequencing traits of longer amplification primers are indicated in the figure.

Wang *et al.* [6] were the first to associate NGS and GW technologies to analyze the insertion sites of the immunodeficiency virus (HIV) into the human genome. In this first approach, authors used a classical restriction-based GW approach, consisting essentially in the digestion of genomic DNA molecules with a restriction enzyme and subsequent ligation of restriction fragments to DNA cassettes. The library of PCR fragments obtained by amplifying the ligation products was directly used for pyrosequencing (Figure 1A). A similar approach, with the improvement of the introduction of biotinylated primers, but also with some complicated and unuseful steps, such as the ligation of a double set of adapters and a primer extension reaction to create an additional restriction site, was used to analyze the transposon flanking sequences in 1,000 petunia *dTph1* insertional mutants [12]. Interestingly, however, in this paper the use of a nucleotide barcode to distinguish specific samples was firstly adopted in NGS-GW experiments. At the same time, an improved version of their first NGS-GW approach, with the inclusion of a DNA barcoding step (Figure 1B) was also published by Wang *et al.* [13]. One hundred and sixty thousand integration sites for lentiviral and gamma-retroviral vectors in twenty-eight tissue samples from eight different mice could be identified by this approach. 

Liu *et al.* [14,15] combined another restriction-based GW approach, known as Digestion Ligation Amplification (DLA) with pyrosequencing. DLA-GW is characterized by the use of a single-stranded oligonucleotide adaptor to ligate to restricted genomic DNA fragments. In this case the introduction of a DNA-barcode was also employed for sequencing in a single experiment multiple independent insertional mutants of the high copy number *Mu* transposon from different maize *Mu*-stocks. The assay allowed the observation of the expected *Mu*/genomic junctions in approximately 94% of the 965,000 reads, demonstrating the specificity of the strategy. 324 gene hotspots for *Mu* insertions were detected.

The first application of the pyrosequencing-GW approach to yeast is by Guo and Levin [16] who studied the integration of the *Tfl* retrotransposon in the genome of *Schizosaccharomyces pombe*. About 600,000 sequences were analyzed, allowing the identification of more than 73,000 independent integration sites.

**Figure 2:**
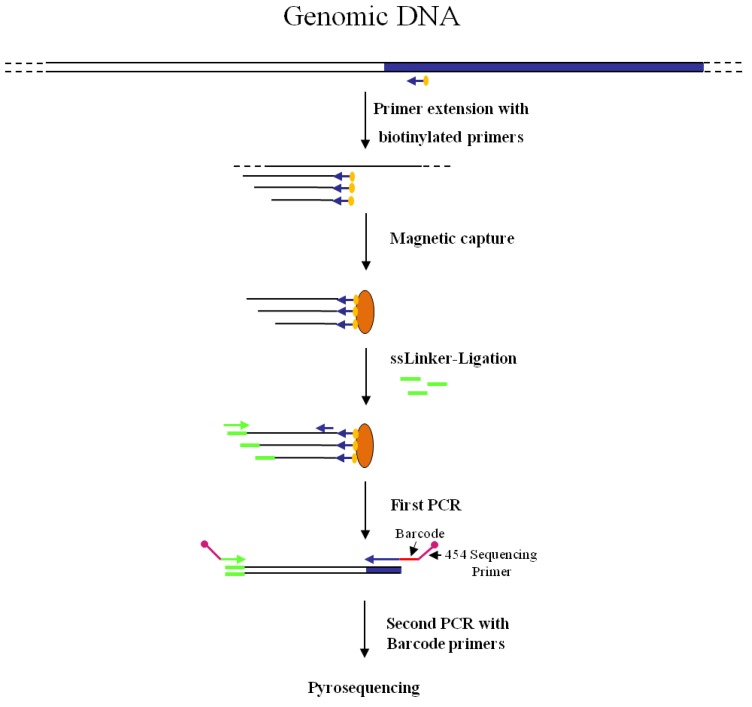
Main steps of nrLAM-PCR GW and pyrosequencing association strategy. Blue and white bars correspond to known and unknown sequences, respectively. A blue arrow with a small yellow oval corresponds to biotinylated primers in the known nucleotide region. An orange oval indicates a streptavidin magnetic device. Green and blue arrows represent primers designed in correspondence of the linker and known genomic region, respectively. Barcodes and 454 sequencing traits of longer amplification primers are indicated in the figure.

The above reported GW-pyrosequencing strategies adopted two restriction-based GW methods. Subsequently pyrosequencing was also applied to the Extension-based GW method known as nonrestrictive Linear Amplification-Mediated PCR (nrLAM-PCR) [9,10]. Extension-based GW methods are based on the extension of an oligonucleotide specific of the known DNA region and directed toward the unknown sequence. After the synthesis of the single-strand DNA, several strategies can be adopted to make it a suitable substrate for PCR reactions (reviewed in [1]). The basic method (known as Ligation Mediated PCR, LM PCR) was firstly introduced in 1989 by Muller and Wold [17,18] and several modifications, including the LAM-PCR, have been added since then. In the original LAM-PCR procedure [19] a 5'-biotynylated primer is extended from the known region of the genomic DNA and then a complementary strand is synthesized on the purified extension product by random exanucleotide priming. The obtained DNA molecule is then digested with a restriction enzyme recognizing a four nucleotide site, and ligated to a DNA cassette for providing the substrate for a final PCR amplification. In the “nonrestrictive” version of the method, the product of the extension reaction is ligated to a specific single-strand linker by means of RNA ligase. The strategy was designed in order to study the critical step of vector DNA integration during gene therapy, as a possible origin of the interruption of important genes and/or activation of proto-oncogenes by vector-introduced promoter and enhancer sequences. Figure 2 shows the combined nrLAM-pyrosequencing strategy, including barcoding of amplicons to facilitate clonal identification of the insertional events.

## 3. GW by Illumina-SBS

**Figure 3:**
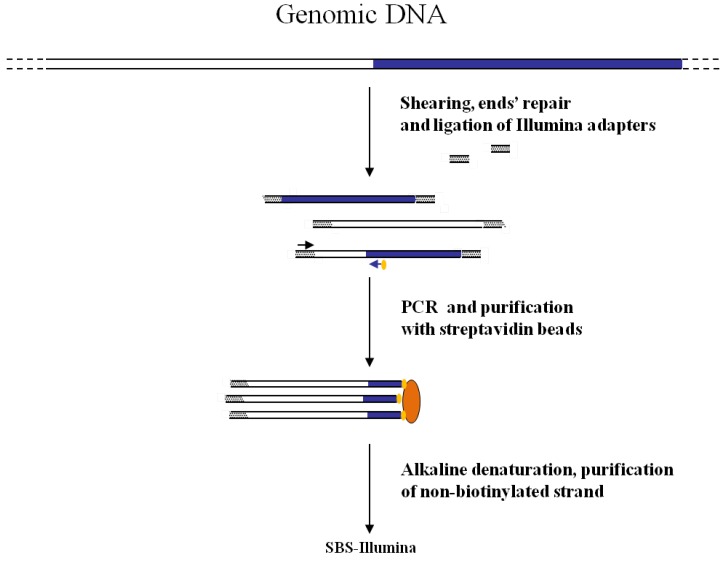
Main steps of cassette-PCR GW and sequencing by synthesis (SBS)-Illumina association strategy. Blue and white bars correspond to known and unknown sequences, respectively. A blue arrow with a small yellow oval corresponds to biotinylated primers in the known nucleotide region. A black horizontal arrow indicates adapter specific primers. An orange oval indicates a streptavidin magnetic device.

The first association of the SBS-Illumina NGS approach to GW was reported by Gawronski *et al.* [7] in their study for the identification of *Haemophilus influenzae* virulence genes required to delay bacterial clearance in the lungs of mice. The assay was carried out by sequencing insertion sites of a *Himar1-mariner* transposon insertional library of *H. influenzae* after infection in lungs of 5 mice (negative selection). Transposon/bacterial DNA junctions were identified in the resulting libraries by the so-called “high-throughput insertion tracking by deep sequencing” (HITS) method, practically consisting in a massive GW analysis by the Illumina technology (Figure 3). In this strategy the fragmentation of genomic DNA by restriction enzymes (as shown in Figure 1) has been replaced by “shearing”. The additional step of repairing DNA ends is however necessary before ligation of adapters. After repairing of the sheared DNA ends from transposon mutant libraries and ligation of Illumina oligonucleotide adapters, transposon-containing fragments are enriched via PCR using a biotinylated transposon primer and affinity purification on streptavidin-coated paramagnetic beads. Purified fragments are then used for sequencing according to Illumina protocols. Using this approach, a large library of approximately 75,000 insertional mutants was analyzed, providing a rapid genome-wide analysis of bacterial genes required for growth/survival during infection of host organisms.

In an almost contemporary paper, a similar approach was used to investigate a large *Tn5*-derived bacterial transposon insertional library produced in *Salmonella enterica* for the identification of interrupted genes [20]. Sequencing was directly carried out on amplicons obtained with one primer corresponding to the transposon, and a second primer corresponding to the Illumina adaptor. Even if not mentioned by the authors, this approach stands as a classical NGS-GW approach. A *Tn5*-derived transposon was used to generate an estimated pool of 1.1 million transposon mutants and 370,000 unique transposon insertion sites were identified. Authors performed also a negative selection in order to identify *S. enterica* genes involved in the bacterium resistance to high concentrations of bile. 169 genes involved in bile tolerance were identified by this approach. 

The same approach was successful for the analysis of an eukaryotic transposon insertional library. Li *et al.* [21] analyzed an inducible *piggyBac* (*PB*) transposon-based mutagenesis library in the yeast *S. pombe*. From a mutant pool of 400,000 Arg+ Ura+ colonies, *PB* insertions were detected in 54% of the ORFs containing the typical TTAA *PB* insertion site.

Illumina-GW proved also effective in the identification of *Mu* transposon insertions in the genome of maize photosynthesis mutants [22]. Distinct DNA samples were pooled and analyzed thanks to the introduction of barcodes. The problem of unambiguous assignment of transposon flanking regions, which may be encountered in the analysis of complex eukaryotic genomes, was addressed by authors by using 60-mer long biotinylated primer and low-cycle PCR amplifications. The approach allowed the identification of four genes whose interruption blocks chloroplast biogenesis. In a similar approach Urbanski *et al.* [23] analyzed a pool of Lotus mutant plants containing the retrotransposon *LORE1*. In this assay, the blunt-ended sheared DNA fragments were first subjected to 3' adenylation by *Taq* polymerase, and then ligated to splinkerette-adaptors provided of single T-overhangs. Thanks to this strategy 3,744 plants were examined and 8,935 new *LORE1* insertion sites were identified.

**Figure 4:**
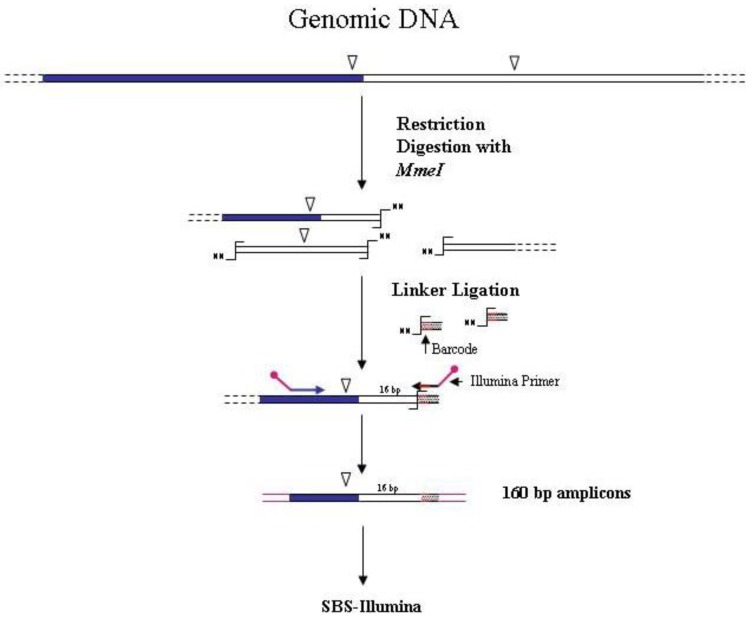
Main steps of *MmeI*-assisted cassette-PCR GW and SBS-Illumina association strategy. Blue and white bars correspond to known and unknown sequences, respectively. An open vertical arrow indicates the *MmeI* recognition site. The 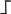 symbol indicates the *MmeI* digestion site. A black horizontal arrow indicates adapter specific primers; a blue arrow represents primers designed in the known region. Barcodes and Illumina sequencing traits of longer amplification primers are indicated in the figure.

A different strategy for sequencing transposon/genomic DNA junctions was ideated by van Opijnen *et al.* [8,24] to identify the insertion sites of the *mariner Himar1* mini-transposon into the genome of the Gram-positive bacterium *Streptococcus pneumoniae*. The strategy is highly innovative and must not be confused with a restriction-based/NGS approach. Indeed in this case, differing from all the restriction-based GW methods, the enzyme recognition site is not on the genomic DNA of the mutated organism, but in the transposon used for mutagenesis. The method relies on the presence of a *MmeI* recognition site at four bp from the left and right ends of the inverted repeats of the transposon. *MmeI* cuts 20 bp downstream of the recognition site and generates a random two-nucleotide 3'-overhang. After restriction of the library DNA, the ligation of an adapter with a random two-nucleotide overhang allows the amplification of unique-size fragments using transposon and adapter specific primers. Amplicons of 160 bp will contain 16 bp of flanking genomic DNA. The introduction of a DNA barcode was also employed for sequencing different samples in a single flow cell lane using the Illumina approach (Figure 4). The *S. pneumoniae* library was analyzed to categorize genes according to their relevance in bacterial fitness, allowing identification of genes essential for basal growth. A similar approach was also used by Goodman *et al.* [25] for the identification of fitness genes of the gut symbiont *Bacteroides thetaiotaomicron*.

Brett *et al.* [26] designed an Illumina-GW procedure for the analysis of *Sleeping Beauty* (*SB*) induced tumors in mice. The method is based on the ligation of adapters to genomic DNA fragments obtained by digestion with restriction enzymes (*AluI* or *NlaIII*). After a first PCR with transposon and adapter specific primers, a nested PCR is performed with primers provided of barcodes and tags for sequencing on Illumina platforms. Regardless of the NGS technology, the approach is similar to that described by Wang *et al.* [13] and illustrated in Figure 1B.

**Figure 5:**
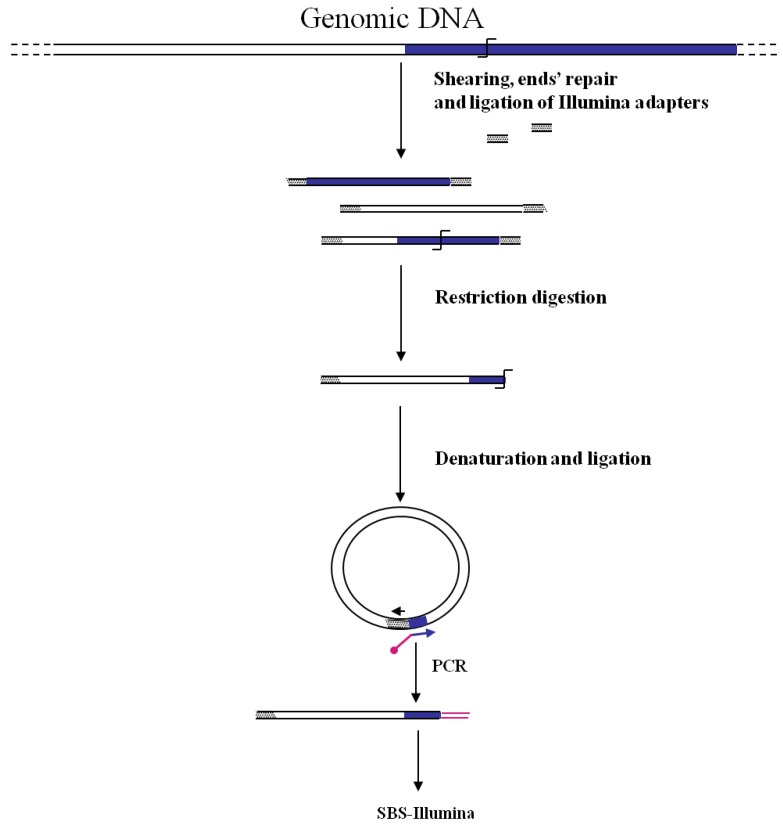
Main steps of Inverted-PCR GW and SBS-Illumina association strategy. Blue and white bars correspond to known and unknown sequences, respectively. An open vertical arrow indicates the *MmeI* recognition site. The 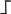 symbol indicates a restriction site. A black horizontal arrow indicates adapter specific primers; a blue arrow represents primers designed in the known region. The Illumina sequencing trait of longer amplification primers is indicated in the figure.

An innovative NGS-GW approach is that reported by Gallagher *et al.* [27] to track large numbers of transposon mutants of *Pseudomonas aeruginosa*. In this case authors associated an inverted-PCR GW approach (firstly described by Triglia *et al.* [28]) to the Illumina NGS technology (Figure 5). Genomic DNA from a mutant pool is sheared, end-repaired and ligated to one Illumina adapter. DNA fragments are then digested by a proper restriction enzyme cutting the transposon near the junction with the unknown DNA. After size-selection, restriction fragments are denaturated and single-strand DNA self-ligated. A second PCR step with divergent primers corresponding to the adaptor and to the transposon allows the identification of transposon/genome junctions. Illumina sequencing can be performed by introducing into the transposon primer the required Illumina adaptor sequence. The procedure was tested by screening a pool of 100,000 mutants for the identification of tobramycin resistance genes. A total of 117 resistance genes were identified, including previously unknown ones. 

## 4. Other Possible NGS-GW Pairing and General Conclusions

The possibility to combine other NGS and GW strategies, in addition to those already reported, is examined in this paragraph. This analysis, besides constituting a preliminary base for future applications, also gives the opportunity to draw some general conclusions about the NGS and GW strategies to combine for optimal results.

Taking into account that all the GW methods have a final PCR amplification step, NGS methods relying on the synthesis of large arrays of amplicons as substrate for sequencing can in principle be combined. Even if currently there are no articles reporting the application of the SOLiD and Ion Torrent NGS technologies to GW methods, the possibility to construct large barcoded libraries has been recently illustrated for both methods, making them eligible for large, multiplex, GW applications. For example, the construction of barcoded libraries for SOLiD sequencer was reported by Farias-Hesson *et al.* [29], who prepared 32 libraries by ligation of end-repaired sheared DNA molecules to SOLiD adaptors, one of which provided with 6-bp barcode. This procedure should be also adaptable to multiple sequence analysis of the same targeted region (*i.e.*, a transposon) amongst several biological samples (see also the Applied Biosystem Application note “SOLiD System Barcoding” [30]). Additional examples of the use of barcoding to analyze the same targeted region in several biological samples by SOLiD technology can be found in some recent papers [31,32,33,34]. The possibility to apply the Ion Torrent sequencing technology to GW can be deduced from the genotyping-by-sequencing approach exemplified for the complex genomes of barley and wheat and available at the Invitrogen website [35]. The lower cost of the Personal Genome Machines by Ion Torrent compared to SOLiD platforms, together with their reduced times for amplicon sequencing, can make them probably preferred for most NGS applications, including GW.

From the analysis of the GW-NGS studies reported in the previous paragraphs, it comes out that pyrosequencing has been exclusively used for eukaryotic genomes, while the SBS-Illumina approach has been adopted for both prokaryotic and eukaryotic genomes. However, a definitive indication about the most appropriate NGS technology to use in GW insertional mutation analysis is not really relevant at this time, since all the NGS approaches reported above have larger sequencing capacity than that needed for identification of insertion sites. This is true even for high-copy transposons in eukaryotic genomes, as the *Mu* transposon in maize, which has been tracked by both GW-pyrosequencing [14,15] and GW-Illumina approaches [22]. Accordingly, in all the cases the introduction of DNA barcodes allowed the analysis of multiple samples in single sequencing experiments. In the case of *SB*-induced tumors, however, Brett *et al.* [26] reported about the higher capacity of insertion sites detection by SBS-Illumina compared to 454-pyrosequencing. Even if some experimental details were different, comparison of the two sequencing strategies in the analysis of ligation-mediated PCR libraries showed an increased sensitivity of the Illumina approach respect to pyrosequencing of about 50 fold, allowing the detection of rare transposon insertion events. In the case of bacteria with high GC-content, Ion Torrent approach has been found more reliable than Illumina, with a more stable quality of sequencing data (reviewed in Liu *et al.* [36]).

Also for the GW method to combine with NGS technologies, data available indicate that both restriction-based and extension-based approaches are suitable for adaptation to NGS technologies. The first case is exemplified by the cassette-ligation (Figure 1), its “shearing-adapted” version (Figure 3), and by the self-ligation (Figure 5) methods. The latter approach is shown in Figure 2. Association of primer-based GW methods to NGS approaches appears more difficult. Primer-based GW strategies start with a preliminary PCR carried out with a primer containing degenerate sequences (possibly annealing in the unknown region of the genome) and a primer corresponding to the known sequence (*i.e.*, a gene region or an insertion sequence). Subsequently PCR products are further selected by hemi-nested PCR amplifications using a second primer specific for the known region. Even if in this last step nested primer containing DNA barcodes and NGS sequencing regions can be designed, making the association to NGS approaches theoretically possible, currently there are no reports about the combined use of the two methods.

Another critical parameter to be considered in the choice of the GW approach is its sensitivity. Some specific comparative studies have been carried out and discussed in a recent review article [1]. Generally speaking, however, the use of solid-phase purification of biotinylated DNA fragments has highly contributed to increasing the sensitivity of GW methods. For example in studies on the integration of foreign DNA into salmon genome, it has been found that classical anchored PCR amplifications, combined with solid-phase purification, allow identification of about two copies of the target sequence in 25 ng of DNA [37]. Several of the above reported GW-NGS approaches include streptavidin purification of biotinylated DNA molecules [9,10,12,22].

Finally, the need of proper bioinformatic resources for the analysis of GW-NGS data cannot be disregarded. A typical analysis pipeline includes: sorting of sequences according to the incorporated barcode for the identification of sequences belonging to individual amplicons; removal of foreign genome sequences (linkers, transposons, vectors); clustering and counting of identical sequences and mapping of insertion sites. Good examples of bioinformatic analysis with description of the employed tools and filtering parameters are given in several articles on GW-NGS applications [7,9,12,14,15,20,21,22,23,26,27].

In conclusion, GW-NGS approaches have the potentiality to make insertional mutagenesis a high throughput screening approach of wide use in functional genomics, replacing more classical methods such as microarray hybridization. In addition, the combined methods showed interesting applications in medicine for the study of the integration mechanisms of retrovirus and DNA vector for gene therapy. 
